# Association of High Normal Body Weight in Youths With Risk of Hypertension

**DOI:** 10.1001/jamanetworkopen.2023.1987

**Published:** 2023-03-14

**Authors:** Corinna Koebnick, Margo A. Sidell, Xia Li, Susan J. Woolford, Beatriz D. Kuizon, Poornima Kunani

**Affiliations:** 1 Department of Research and Evaluation, Kaiser Permanente Southern California, Pasadena; 2 Child Health Evaluation and Research Center, Department of Pediatrics, University of Michigan, Ann Arbor; 3 Kaiser Permanente Los Angeles Medical Center, Los Angeles, California; 4 Department of Pediatrics, Kaiser Permanente Manhattan Beach Medical Office, Manhattan Beach, California

## Abstract

**Importance:**

Ample evidence links obesity to hypertension in youths. However, the association of high normal body mass index (BMI) with obesity and the interaction with different weight trajectories are not well understood.

**Objective:**

To examine the hypertension risk associated with high normal BMI for age and different weight trajectories in youths.

**Design, Setting, and Participants:**

This retrospective cohort study assessed 801 019 youths aged 3 to 17 years in an integrated health care system in Southern California from January 1, 2008, to February 28, 2015, with a maximum follow-up of 5 years from January 1, 2008, to February 28, 2020. Data analysis was performed from 2018 to 2022.

**Exposures:**

Youths were compared by first available (baseline) sex-specific BMI for age and change in the distance to the median BMI for age during the 5-year follow-up.

**Main Outcomes and Measures:**

Cox proportional hazards regression models with age as a time scale to assess hypertension risk (based on 2017 Blood Pressure Guidelines by the American Academy of Pediatrics from 3 consecutive independent visits), adjusted for sex, race and ethnicity, socioeconomic status, baseline year, and birth year.

**Results:**

A total of 801 019 youths (mean [SD] age, 9.4 [4.6] years; 409 167 [51.1%] female]; 59 399 [7.4%] Asian and Pacific Islanders, 65 712 [8.2%] Black, and 427 492 [53.4%] Hispanic) were studied. Compared with youths with a baseline BMI for age in the 40th to 59th percentiles, the adjusted hazard ratio (aHR) for hypertension within a maximum of 5 years was 1.26 (95% CI, 1.20-1.33) for youths between the 60th and 84th percentiles if they maintained their BMI for age. With every 1-unit annual increase in the distance to the median BMI for age, the aHR increased by 1.04 (95% CI, 1.04-1.05). The aHR was 4.94 (95% CI, 4.72-5.18) in youths with a baseline BMI for age in the 97th percentile or higher who maintained their body weight. Weight gain increased the risk associated with baseline BMI for age in the 97th percentile or higher with an aHR of 1.04 (95% CI, 1.04-1.05) per 1-unit annual increase in the distance to the median BMI for age. The risk associated with weight change was higher in youths living with low to high normal weight and overweight than in youths living with severe obesity.

**Conclusions and Relevance:**

In this cohort study of youths, high normal body weight above the 60th percentile of BMI for age was associated with increased risk of hypertension. Weight gain was associated with further increases in hypertension risk. Further research is needed to evaluate the wide range of body weight considered normal in youths and the health risks associated with high normal weight.

## Key Points

**Question:**

How are body weight and change in body weight over time associated with the risk of hypertension in youths at the higher end of normal weight?

**Findings:**

In this cohort study including more than 800 000 youths, weight change expressed as a change in the distance from the median BMI for age per year was associated with a change in hypertension risk independent of baseline weight classes. However, the risk associated with weight change was higher in youths living with low to high normal weight and overweight than in youths living with severe obesity, indicating a plateau in the association between weight gain and risk of hypertension.

**Meaning:**

These findings suggest that even modest elevations in the BMI-for-age percentile in the upper range of normal weight may confer an increased risk of hypertension among children, which may further increases with excess weight gain over time.

## Teaser

This cohort study of youths in southern California aged 3 to 17 years explores the risk of hypertension associated with high normal weight at baseline and differentiates between the risk of hypertension associated with baseline body weight and the risk associated with additional gain in body weight over time.

## Introduction

In 2015 to 2018, the prevalence of hypertension was 4.6% in US children aged 8 to 12 years and 3.7% in US adolescents aged 13 to 17 years.^1^ Hypertension during youth tracks into adulthood and is associated with cardiac and vascular target organ damage, such as thickening of the arteries, increased arterial stiffness, and decreased endothelial function.^2^ With increasing evidence that the target organ damage might become irreversible independent of blood pressure control,^3,4^ preventing sustained hypertension and associated target organ damage in children is essential. Obesity may be the most potent modifiable risk factor for hypertension during childhood.^5,6,7,8,9,10,11^

The epidemiologic evidence linking childhood obesity to the risk of hypertension is substantial.^1,8,10,12,13,14^ US data indicate a prevalence ratio for hypertension of 1.54 (95% CI, 0.64-3.69) for children who are overweight and 3.05 (95% CI, 1.78-5.20) for children with obesity.^1^ Hypertension increases disproportionally in youths who are severely obese with a sex- and age-specific body mass index (BMI; calculated as weight in kilograms divided by height in meters squared) above the 97th percentile.^15^ However, 2 current knowledge gaps exist. First, normal body weight in youths has a wide range from the 5th to the 85th percentiles of BMI for age,^16,17,18^ and, to our knowledge, no data exist to assess the health risk associated with low or high normal body weight. Second, data are lacking to differentiate between the risk of hypertension during childhood associated with baseline body weight and the risk associated with additional gain in body weight over time. With an increasing proportion of US youths who are heavier than reference youths based on Centers for Disease Control and Prevention (CDC) growth charts,^17,18^ the determination of the risk associated with excess body weight and weight gain has clinical and public health implications for strategies to prevent and treat pediatric hypertension.

We designed the current study to (1) assess the risk of hypertension associated with high normal weight at baseline and (2) differentiate between the risk of hypertension associated with baseline body weight and the risk associated with additional gain in body weight over time. For the current study, we divided normal body weight into low (5th-39th percentiles), medium (40th-59th percentiles), and high (60th-84th percentiles) to provide insight into the risk of hypertension at a weight below the threshold for overweight. We report the incidence of hypertension within 5 years from a large multiethnic cohort of youths in southern California between 3 and 17 years of age who were passively followed up through electronic medical records.

## Methods

### Study Design and Participants

For the current retrospective cohort study, we identified youths between the ages of 3 and 17 years at baseline who were actively enrolled in a health plan between January 1, 2008, and February 28, 2015, with Kaiser Permanente Southern California (KPSC), a large, prepaid, integrated managed health care system.^19^ The follow-up of the cohort until the end of the study on February 28, 2020, was conducted through passive surveillance of clinical care information using an electronic health record (EHR) system. The first available blood pressure during the study period was used as the baseline date. Youths were ineligible to participate if they had no blood pressure or body weight measurements, preexisting hypertension, medical conditions known to significantly affect growth or blood pressure, or any chronic complex care conditions (eFigure in Supplement 1).^20,21^ We identified 940 409 eligible youths who were free of hypertension. We then excluded 139 390 youths with missing follow-up information because they left the KPSC health plan, gaps (>90 days) in health insurance coverage, missing valid follow-up blood pressures (blood pressures taken during medical visits indicating fever or pregnancy were not considered valid), or missing follow-up height to calculate blood pressure percentiles. The final analytical cohort comprised 801 019 hypertension-free youths who had their index date defined as the first visit with a BMI between 2008 and 2015 and at least 1 follow-up blood pressure within 5 years of the defined index date. The study protocol was reviewed and approved by the institutional review board of KPSC. The requirement of informed consent was waived. All data were deidentified. The study followed the Strengthening the Reporting of Observational Studies in Epidemiology (STROBE) reporting guideline.^22^

### Blood Pressure Measurements and Classification

Blood pressure was measured routinely at the beginning of almost every outpatient clinical visit, as described in detail elsewhere.^15^ Nurses and medical assistants were trained following the guidelines of the American Association of Critical Care Nurses for pediatric care.^23^ Digital devices (Welch Allyn Connex series, Welch Allyn Inc) are the preferred blood pressure measurement devices at KPSC. In some cases, a wall-mounted or portable aneroid sphygmomanometer (Welch Allyn Inc) was used. A full range of different cuff sizes was available at the locations where vital signs, including blood pressure, were measured in the clinics. All staff measuring blood pressure were certified in blood pressure measurement during their initial staff orientation and recertified annually.

Blood pressure measures for all outpatient encounters were extracted from the EHR unless the measured body temperature at the time of the encounter was greater than 38.0 °C. We calculated blood pressure percentiles for age, sex, and height using 2017 updated normative tables and definitions based on children with normal body weight by the American Academy of Pediatrics.^24^ In youths between the ages of 3 and younger than 13 years, blood pressure was defined as stage I hypertension if at the 95th percentile or greater to less than the 95th percentile plus 12 mm Hg (or ≥130/80 mm Hg, whichever is lower) and as stage II hypertension if at the 95th percentile plus 12 mm Hg (or ≥140/90 mm Hg, whichever is lower).^24^ For youths 13 years or older, blood pressure was defined as stage I hypertension if 130/80 to 139/89 mm Hg and as stage II hypertension if 140/90 mm Hg or higher.^24^

### Definition of Study Outcome

The primary study outcome was incident and persistent hypertension. During follow-up, participants were classified as having hypertension if they met 1 of the following conditions: (1) stage I hypertension confirmed during 3 independent medical visits, (2) stage II hypertension confirmed in at least 1 additional independent visit as stage II hypertension or higher, (3) 2 medical visits with a diagnosis of hypertension (defined as *International Classification of Diseases, Ninth Revision [ICD-9]* codes 401-405 and 362.11; *International Statistical Classification of Diseases and Related Health Problems, Tenth Revision [ICD-10]* codes I10-I13, I15.0, I15.8, and H35.039), or (4) 1 diagnosis of hypertension and a prescription of antihypertensive drugs.^24,25^ The date of the first occurrence was used as the event date. If the conditions above were not met (eg, a single high blood pressure), the occurrence was not counted as an event. For a sensitivity analysis, we used any first occurrence as an event: a single blood pressure indicating stage I or II hypertension and a single medical visit with a diagnosis of hypertension.

### Cohort Follow-up

Youths were followed up passively for a maximum of 5 years through February 28, 2020, using information extracted from the EHR. We calculated the follow-up time from a child’s index date until the first occurrence of 1 of the following events: incidence of hypertension, pregnancy, end of KPSC health care coverage, death, end of follow-up on February 28, 2020, or 5 years after the index date.

### Body Weight and Height

Body weight and height were routinely measured and extracted from the EHR. Biologically implausible BMI was defined as upper and lower 0.01 percentile (lower BMI of 7.9 and upper BMI of 70.8) and omitted before analysis (n = 5912 BMIs of 28 804 620 measurements). Definitions of overweight and obesity in children and adolescents are based on the sex-specific BMI-for-age growth charts developed by the CDC.^16^ Youths were categorized as underweight (BMI for age <5th percentile), low normal weight (BMI for age ≥5th to <40th percentiles), medium normal weight (BMI for age ≥40th to <60th percentiles), high normal weight (BMI for age ≥60th to <85th percentiles), overweight (BMI for age ≥85th to <95th percentiles), moderately obese (BMI for age ≥95th to <97th percentiles), and severely obese (BMI for age ≥97th percentile). Change in BMI from baseline was calculated for each follow-up visit as absolute difference in the distance from the median BMI for age and sex.^26^

### Covariates

We obtained sex, age, and year of birth from EHRs. Data for this study included youths whose parents reported their race/ethnicity as Asian or Pacific Islander, non-Hispanic Black or African American (hereafter, Black), non-Hispanic White (hereafter, White), Hispanic or Latino (regardless of race, hereafter, Hispanic), and other or unknown race or ethnicity based on various sources, such as registration records, clinical visit records, and birth certificates. The category *other* includes self-reported “other races or ethnicities” and “multiple races or ethnicities.” Data on race and ethnicity were obtained to investigate whether any racial and ethnic patient populations experienced disparities in their health and health care. We used government assistance for health care insurance (yes/no), such as Medicaid, as a proxy for a low socioeconomic status.

### Statistical Analysis

Data analysis was performed from 2018 to 2022. Baseline characteristics, including sex, race and ethnicity, and socioeconomic status, were summarized as mean (SD) or numbers (percentages) reported by baseline BMI-for-age category. Incidence rates (IRs) of hypertension were estimated by dividing the number of hypertension cases by the total person-years of follow-up, overall and within each subgroup, reported as IR per 1000 person-years. The occurrences of hypertension followed a Poisson distribution, and 95% CIs were estimated accordingly. To assess the association between baseline BMI for age, absolute change in the distance from median BMI for age, and risk of hypertension, we used Cox proportional hazards regression models with age (in years) as the time scale and birth year (in 5-year intervals, eg, 2000-2004) as strata. Sex, race and ethnicity, state-subsidized health insurance, and BMI-for-age category were treated as fixed effects; absolute change in the distance from the median BMI for age was modeled as a time-varying factor and can be interpreted as change per year (with age as the time scale of the model). We first modeled the effect of BMI-for-age class at baseline with and without adjusting for the change in distance from the median BMI for age. Next, we tested interaction terms in the fully adjusted model between change in distance from the median BMI for age and sex, state-subsidized health insurance, and baseline BMI-for-age category. We determined the best model fit based on the lowest Akaike information criterion value. Our final model included a 2-way interaction (change in distance from the median BMI for age × baseline BMI-for-age category). Adjusted hazard ratios (aHRs), corresponding 95% CIs, and *P* values for interactions were reported. Sensitivity analysis using only 1 hypertensive blood pressure to define the outcome of hypertension did not result in relevant changes of the risk estimates. All *P* values were 2-sided, and *P* < .05 was considered significant. All statistical analyses were conducted using SAS software, version 9.4 (SAS Institute Inc).

## Results

### Cohort Demographic Characteristics and Crude Hypertension IRs

A total of 801 019 youths (mean [SD] age, 9.4 [4.6] years; 409 167 [51.1%] female]; 59 399 [7.4%] Asian and Pacific Islanders, 65 712 [8.2%[ Black, and 427 492 [53.4%] Hispanic) were studied (Table). During 3 579 994 person-years of follow-up with (mean [SD] follow-up time, 4.47 [1.20] years; mean [SD] number of office visits with blood pressure measurement, 9.9 [7.4]), we identified 24 969 youths with incident hypertension (IR, 6.97 per 1000 person-years; 95% CI, 6.89-7.06) (eTable 1 in Supplement 1). The cohort contributed a total of 7 898 716 data points to the analysis of which 5 815 025 (74.6%) had blood pressure assessments. The IRs per 1000 person-years were higher among boys (8.49; 95% CI, 8.36-8.63) compared with girls (5.52; 95% CI, 5.42-5.63), youths with a state-subsidized health plan (7.91; 95% CI, 7.72-8.11) compared with those without (6.70; 95% CI, 6.61-6.80), and highest among White (7.20; 95% CI, 7.02-7.38) and Hispanic youths (7.19; 95% CI, 7.08-7.32) compared with other youths (IRs ranged from 5.71 to 6.40). The IRs were similar for youths who were underweight and had low normal weight but then increased gradually with increasing BMI-for-age category.

**Table.** Baseline Characteristics of Youths by Baseline BMI for Age^a^

Characteristic	Baseline BMI-for-age percentile							Total
	Underweight (<5th)	Low normal (5th-39th)	Medium normal (40th-59th)	High normal (60th-84th)	Overweight (85th-94th)	Moderate obesity (95th-96th)	Severe obesity (≥97th)	
Sample sizes	25 880 (3.2)	166 859 (20.8)	128 176 (16.0)	215 993 (27.0)	132 464 (16.5)	41 465 (5.2)	90 182 (11.3)	801 019 (100.0)
Sex								
Male	12 916 (49.9)	82 057 (49.2)	61 603 (48.1)	101 367 (46.9)	62 447 (47.1)	20 793 (50.1)	50 669 (56.2)	391 852 (48.9)
Female	12 964 (50.1)	84 802 (50.8)	66 573 (51.9)	114 626 (53.1)	70 017 (52.9)	20 672 (49.9)	39 513 (43.8)	409 167 (51.1)
Age at index, mean (SD), y	7.9 (4.5)	8.8 (4.7)	9.2 (4.7)	9.5 (4.7)	10.0 (4.5)	10.4 (4.2)	9.9 (4.3)	9.4 (4.6)
Race and ethnicity^b^								
Asian or Pacific Islander	3498 (13.5)	17 335 (10.4)	10 625 (8.3)	14 948 (6.9)	7530 (5.7)	2022 (4.9)	3441 (3.8)	59 399 (7.4)
Black	2067 (8.0)	13 020 (7.8)	10 440 (8.1)	18 518 (8.6)	10 777 (8.1)	3248 (7.8)	7642 (8.5)	65 712 (8.2)
Hispanic	11 018 (42.6)	75 862 (45.5)	62 508 (48.8)	113 562 (52.6)	77 716 (58.7)	26 069 (62.9)	60 757 (67.4)	427 492 (53.4)
White	7388 (28.5)	49 229 (29.5)	35 966 (28.1)	55 068 (25.5)	28 374 (21.4)	7638 (18.4)	13 317 (14.8)	196 980 (24.6)
Other or unknown^c^	1909 (7.4)	11 413 (6.8)	8637 (6.7)	13 897 (6.4)	8067 (6.1)	2488 (6.0)	5025 (5.6)	51 436 (6.4)
State-subsidized health plan								
No	20 601 (79.6)	131 975 (79.1)	100 330 (78.3)	166 132 (76.9)	99 472 (75.1)	30 514 (73.6)	65 261 (72.4)	614 285 (76.7)
Yes	5279 (20.4)	34 884 (20.9)	27 846 (21.7)	49 861 (23.1)	32 992 (24.9)	10 951 (26.4)	24 921 (27.6)	186 734 (23.3)
Blood pressure, mean (SD), mm Hg								
Systolic	96.9 (10.1)	99.1 (10.6)	100.5 (10.9)	101.9 (11.0)	104.2 (11.1)	106.2 (10.9)	106.9 (11.1)	102.1 (11.2)
Diastolic	58.0 (8.1)	58.5 (8.2)	58.8 (8.2)	59.3 (8.2)	60.2 (8.2)	61.2 (8.2)	61.5 (8.3)	59.5 (8.3)
Distance from median BMI for age and sex, mean (SD)								
Absolute	−5.4 (1.5)	−2.1 (0.9)	0.0 (0.5)	2.4 (1.0)	6.0 (1.3)	9.2 (1.2)	15.2 (5.2)	3.2 (5.8)
Relative, %	−24.1 (6.8)	−9.5 (4.1)	0.2 (2.1)	10.8 (4.4)	26.8 (5.9)	41.4 (6.0)	67.8 (23.2)	14.4 (25.8)

Abbreviation: BMI, body mass index (calculated as weight in kilograms divided by height in meters squared).

^a^ Data are presented as number (percentage) of youths unless otherwise indicated.

^b^ Race and ethnicity reported by parents was classified as Asian or Pacific Islander, non-Hispanic Black or African American (ie, Black), Hispanic or Latino (regardless of race; ie, Hispanic), non-Hispanic White (ie, White), and other or unknown race or ethnicity based on various sources such as registration records, clinical visit records, and birth certificates.

^c^ The *other* category includes self-reported “other races or ethnicities” and “multiple races or ethnicities.”

### Baseline BMI for Age and Risk of Hypertension

Youths at or above high normal weight had a higher risk of hypertension than youths with medium normal weight, independent of change in body weight during follow-up. In youths with high normal weight who maintained their BMI for age (Δ distance from median BMI for age during follow-up was 0), the aHR for incidence of hypertension was 1.26 (95% CI, 1.20-1.33), after adjusting for race and ethnicity, state-subsidized insurance coverage, and birth year (Figure). The risk of hypertension increased with increasing baseline weight class. The aHR for hypertension was 4.94 (95% CI, 4.72-5.18) for youths with a BMI for age in the 97th percentile or higher who maintained their BMI for age. Increased baseline weight was associated with a higher risk of hypertension regardless of weight gain and was consistent among youths with stable weight and those who lost or gained weight (eTable 2 in Supplement 1). Similarly, high normal weight was associated with a higher risk of hypertension in children who gained or lost weight compared with their peers with medium normal weight.

**Figure.**
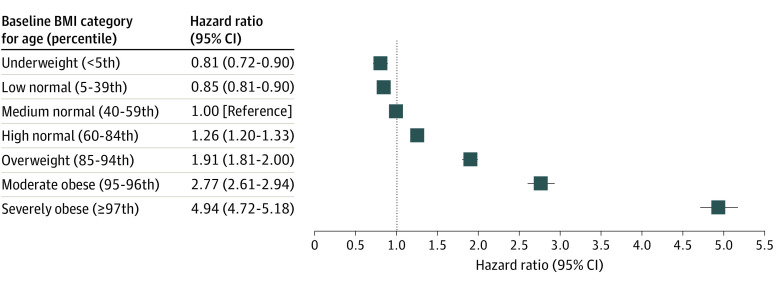
Adjusted Hazard Ratios for Incidence of Hypertension in Youths Aged 3 to 17 Years by Baseline Body Mass Index (BMI) for Age Class If the Distance to the Median BMI for Age Was Maintained Hazard ratios were adjusted for change in distance to the median BMI for age, sex, race and ethnicity, and state-subsidized health plan. Maintaining BMI for age was defined as a 0-unit change in the distance from the median BMI for age at a measurement point from the baseline. BMI was calculated as weight in kilograms divided by height in meters squared.

### Changes in BMI During Follow-up and Risk of Hypertension

Weight change expressed as a change in the distance from the median BMI for age per year was associated with a change in hypertension risk independent of baseline weight classes. However, the risk associated with weight change was higher in youths living with low to high normal weight and overweight than in youths living with severe obesity, indicating a plateau in the association between weight gain and risk of hypertension. The aHR per 1-unit change in the distance from the median BMI for age per year was 1.05 (95% CI, 1.03-1.07) for youths living with a BMI for age in the less than 5th percentile, 1.07 (95% CI, 1.06-1.08) for youths living with a BMI for age between the 5th and 39th percentiles, 1.08 (95% CI, 1.07-1.08) for youths living with a BMI for age between the 40th and 59th percentiles, 1.08 (95% CI, 1.07-1.08) for youths living with a BMI for age between the 60th and 84th percentiles, 1.09 (95% CI, 1.08-1.09) for youths living with a BMI for age between the 85th and 94th percentiles, 1.06 (95% CI, 1.05-1.07) for youths living with a BMI for age between the 95th and 96th percentiles, and 1.04 (95% CI, 1.04-1.05) for youths living withs a BMI for age in the 97th percentile or greater. Although higher baseline body weight is associated with higher hypertension risk at any level of weight gain (eTable 2 in Supplement 1), the additional risk associated with a per 1-unit change in the distance from the median BMI for age per year was lower for youths living with a BMI for age in the 97th percentile or greater.

## Discussion

In this large, prospective cohort study of 801 019 youths, a baseline body weight in the upper range of normal weight (60th-84th percentiles of BMI for age) was associated with an increased hypertension risk compared with youths between the 40th and 59th percentiles of BMI for age, with a 26% higher risk of hypertension if their body weight remained stable during follow-up. Our results suggest that the current range of normal weight from the 5th to 84th percentiles of BMI for age in children may be too wide and requires reevaluation with regard to health risks.

Obesity has been discussed as the main driver for pediatric hypertension during the last few decades.^5,8,10,27^ The prevalence of hypertension is currently approximately 3% in youths with normal weight, 5% in youths who are overweight, and nearly 10% in youths who have severe obesity.^1^ Although several studies^8,10,15,28,29,30,31,32^ have shown that the risk of hypertension is higher in youths with overweight and obesity, information is lacking on high normal weight in youths as a hypertension risk factor. A recent meta-analysis^33^ in adults showed a continuous dose-dependent association between BMI and risk of hypertension starting within the normal BMI range. Our study also suggests that a high normal weight below the threshold for overweight is associated with increased hypertension risk in youths.

Weight gain is associated with hypertension risk in adults.^33^ In that meta-analysis study,^33^ weight gain was associated with a worse prognosis among men than among women. In a prospective cohort study^34^ of Ethiopian children, weight gain from 48 to 60 months and weight at 60 months were associated with hypertension risk. In children with obesity, further weight gain and failure to reduce body weight were associated with higher blood pressure and a higher risk of developing hypertension compared with weight loss after initiation of obesity treatment.^35^ Consistent with other studies,^34,35^ weight gain (defined as an increase in the distance to the median BMI for age) in the current study accelerated and weight loss attenuated the hypertension risk associated with body weight at baseline. As expected, in youth with obesity (BMI for age ≥95th percentile), the escalation or attenuation per 1-unit change in the distance to the median BMI for age was less pronounced than in youths who lived with normal weight.

Weight gain and obesity have complex physiologic sequelae mediated through a decrease in insulin sensitivity and the development of insulin resistance.^36^ The close association between body weight and insulin resistance is partially mediated through inflammatory pathways.^36,37,38,39^ Weight gain and obesity cause changes in the release of adipokines and cytokines from adipose tissue that manifest in metabolic dysfunctions and lead to compensatory hyperinsulinemia, which may increase blood pressure via multiple mechanisms, including inappropriate activation of the renin-angiotensin-aldosterone system.^40,41,42^ The metabolic dysfunction also promotes further weight gain, resulting in a vicious cycle of worsening insulin resistance and its metabolic sequelae.^43^ The dose-dependent association between excess body weight and risk of hypertension in youths described in the current study indicates that obesity-related etiologic pathways leading to hypertension may be activated at an early age and at much lower body weight than suggested by current thresholds for normal weight in youths.

### Strengths and Limitations

Our study has several strengths and limitations. Strengths of the study include EHR data from a large, community-based population with a mean follow-up of more than 4 years; the systematic screening of weight, height, and blood pressure at almost every visit; and information available for potential key confounders and covariates. In addition, the study population is generally reflective of Southern California and includes a high proportion of children born to low-income families.^44^ The cohort study design reduced the chance of possible bias inherent in case-control and hospital-based studies, and the design accounted for factors associated with baseline weight under the assumption of stable weight, weight gain, or weight loss. In addition, the study benefitted from the high frequency of blood pressure and BMI measures throughout childhood, a high quality of weight and height measured due to rigorous training,^45^ and decision support tools to minimize missing data.^46^ The large sample size enabled evaluation of smaller weight categories, including 3 categories for low, medium, and high normal weight, with high precision and allowed us to estimate 2-way interactions between baseline weight and weight over time. This approach allowed us to estimate the association with weight under the assumption of stable weight over time. Limitations include the possibility of residual confounding inherent to the observational design, including the possibility of differential distribution of unmeasured or incompletely measured confounders. We can also not exclude limitations in the generalizability of the results in this California cohort with access to health care and the existence of selection bias.

## Conclusions

The results of this cohort study indicate a strong association between body weight and the risk of hypertension. A high normal body weight in children, ranging from the 60th to the 84th percentile of BMI for age, was associated with increased hypertension risk. Furthermore, the risk of hypertension increased as additional weight gain occurred over time. Under this evidence, further research should reevaluate the current wide range of body weight considered normal and related health risks of high normal weight.

## Notes

Supplement 1. **eTable 1.** Crude Incidence Rate of Hypertension per 1,000 Person-Years by Defined Baseline Covariates **eTable 2.** Change in BMI and Risk of Hypertension in Youth by Baseline Weight Class **eFigure.** Study Flow Chart

Supplement 2.
Data Sharing Statement
