# Supplementary material for: Association of High Normal Body Weight in Youths With Risk of Hypertension
Source: JAMA Netw Open. 2023 Mar 14;6(3):e231987. doi: 10.1001/jamanetworkopen.2023.1987 (PMC10015306; doi:10.1001/jamanetworkopen.2023.1987)
Supplement: Supplement 1. — eTable 1. Crude Incidence Rate of Hypertension per 1,000 Person-Years by Defined Baseline Covariates eTable 2. Change in BMI and Risk of Hypertension in Youth by Baseline Weight Class eFigure. Study Flow Chart [file jamanetwopen-e231987-s001.pdf]

Supplementary Online Content

Koebnick C, Sidell MA, Li X, Woolford SJ, Kuizon BD, Kunani P. Association of high normal body weight in youths with risk of hypertension. *JAMA Netw Open*. 2023;6(3):e231987. doi:10.1001/jamanetworkopen.2023.1987

eTable 1. Crude incidence rate of hypertension per 1,000 person-years by defined baseline covariates

eTable 2. Change in BMI and risk of hypertension in youth by baseline weight class

eFigure. Study flow chart

This supplementary material has been provided by the authors to give readers additional information about their work.

© 2023 Koebnick C et al. *JAMA Network Open.*

eTable 1. Crude incidence rate of hypertension per 1,000 person-years by defined baseline covariates

	Total population	Events	Person-years	Incidence rate per 1,000 person-years (95% CI)
**Overall**	801,019	24,969	3,579,994	6.97 (6.89-7.06)
**Sex**				
Male	391,852	14,869	1,751,232	8.49 (8.36-8.63)
Female	409,167	10,100	1,828,762	5.52 (5.42-5.63)
**Age at Index (yrs.)**				
3-5	263,100	10,442	1,187,282	8.79 (8.63-8.97)
6-11	255,871	7,649	1,156,354	6.61 (6.47-6.76)
12-17	282,048	6,878	1,236,359	5.56 (5.43-5.70)
**Race/Ethnicity**				
API	59,399	1,702	273,238	6.23 (5.94-6.53)
Black	65,712	1,930	301,698	6.40 (6.12-6.69)
Hispanic	427,492	13,895	1,931,254	7.19 (7.08-7.32)
White	196,980	6,344	881,508	7.20 (7.02-7.38)
Other/Unknown	51,436	1,098	192,297	5.71 (5.38-6.06)
**State-subsidized health plan**				
No	614,285	18,560	2,769,697	6.70 (6.61-6.80)
Yes	186,734	6,409	810,298	7.91 (7.72-8.11)
**BMI-for age percentile**				
Underweight (<5th)	25,880	474	117,097	4.05 (3.70-4.43)
Low normal (5-39th)	166,859	2,911	754,873	3.86 (3.72-4.00)
Medium normal (40-59th)	128,176	2,506	577,384	4.34 (4.17-4.51)
High normal (60-84th)	215,993	5,160	969,930	5.32 (5.18-5.47)
Overweight (85-94th)	132,464	4,498	589,191	7.63 (7.41-7.86)
Moderate obesity (95-96th)	41,465	1,908	183,287	10.41 (9.95-10.89)
Severe obesity (≥97th)	90,182	7,512	388,232	19.35 (18.92-19.79)

Acronyms: API, Asian and Pacific Islanders, BMI Body mass index
Race and ethnicity reported by parents was classified as Asian or Pacific Islander (API), non-Hispanic Black or African American (hereafter, Black), Hispanic, or Latino (regardless of race, hereafter, Hispanic), non-Hispanic White (hereafter, White), and other or unknown race/ethnicity based on various sources such as registration records, clinical visit records, and birth certificates. The category “other” includes self-reported category “other races or ethnicities” and “multiple races or ethnicities.

© 2023 Koebnick C et al. *JAMA Network Open.*

eTable 2: Change in BMI and risk of hypertension in youth by sex and baseline weight class

BMI-for-age class at baseline	HR (95% CI)*
**Δ Distance from median BMI-for-age =-10 kg/m²**	
Underweight (<5th)	1.04 (0.76 ,1.40)
Low normal (5-39th)	0.91 (0.78 ,1.07)
Medium normal (40-59th)	1.00 (Reference)
High normal (60-84th)	1.22 (1.08 ,1.38)
Overweight (85-94th)	1.73 (1.52 ,1.97)
Moderate obesity (95-96th)	3.25 (2.83 ,3.74)
Severe obesity (≥97th)	6.57 (5.83 ,7.39)
**Δ Distance from median BMI-for-age =-5 kg/m²**	
Underweight (<5th)	0.91 (0.75 ,1.11)
Low normal (5-39th)	0.88 (0.80 ,0.97)
Medium normal (40-59th)	1.00 (Reference)
High normal (60-84th)	1.24 (1.15 ,1.34)
Overweight (85-94th)	1.82 (1.67 ,1.97)
Moderate obesity (95-96th)	3.00 (2.74 ,3.28)
Severe obesity (≥97th)	5.70 (5.29 ,6.14)
**Δ Distance from median BMI-for-age = 0 kg/m²**	
Underweight (<5th)	0.81 (0.72 ,0.90)
Low normal (5-39th)	0.85 (0.81 ,0.90)
Medium normal (40-59th)	1.00 (Reference)
High normal (60-84th)	1.26 (1.20 ,1.33)
Overweight (85-94th)	1.91 (1.81 ,2.00)
Moderate obesity (95-96th)	2.77 (2.61 ,2.94)
Severe obesity (≥97th)	4.94 (4.72 ,5.18)
**Δ Distance from median BMI-for-age = +5 kg/m²**	
Underweight (<5th)	0.71 (0.63 ,0.81)
Low normal (5-39th)	0.82 (0.76 ,0.89)
Medium normal (40-59th)	1.00 (Reference)
High normal (60-84th)	1.28 (1.20 ,1.37)
Overweight (85-94th)	2.00 (1.87 ,2.14)
Moderate obesity (95-96th)	2.55 (2.36 ,2.76)
Severe obesity (≥97th)	4.29 (4.02 ,4.57)
**Δ Distance from median BMI-for-age = +10 kg/m²**	
Underweight (<5th)	0.63 (0.51 ,0.78)
Low normal (5-39th)	0.79 (0.70 ,0.90)
Medium normal (40-59th)	1.00 (Reference)
High normal (60-84th)	1.31 (1.17 ,1.45)
Overweight (85-94th)	2.10 (1.88 ,2.35)
Moderate obesity (95-96th)	2.36 (2.08 ,2.67)
Severe obesity (≥97th)	3.72 (3.35 ,4.13)

*Adjusted for race/ethnicity, and state-subsidized insurance coverage, and birth year; 2-way interaction baseline BMI-for-age x weight change P < 0.001.

© 2023 Koebnick C et al. JAMA Network Open.

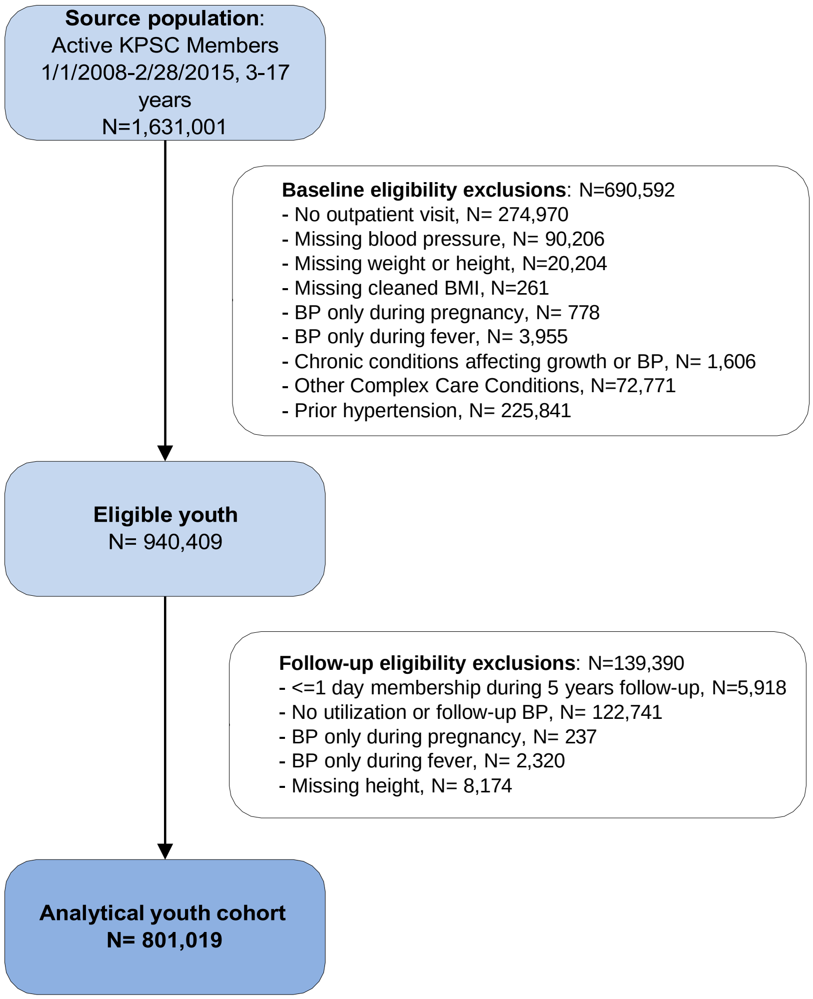

eFigure 1: Study flow chart of KPSC youth at ages 3-17 years with baseline in 2008 through 2015.

BP, blood pressure

Pre-existing hypertension was defined as hypertensive blood pressure ≥95th percentile or a diagnosis of hypertension.

* Medical conditions or chronic conditions known to significantly affect growth or blood pressure: growth hormone deficiency, ICD9 253.3, ICD10 E23.0; overproduction, ICD9 253.0, ICD10 E22.0; aortic coarctation, ICD9 747.10, ICD10 Q25.1; chronic renal disease, ICD9 585, ICD10 N18; congenital adrenal hyperplasia , ICD9 255.2, ICD10 E25; Cushing syndrome, ICD9 255.0, ICD10 E24.0, E24.2, E24.3, E24.8, E24.9; hyperaldosteronism, ICD9 255.1, ICD10 E26; hyperthyroidism, ICD9 242, ICD10 E05.

Complex chronic conditions are any medical condition that can be reasonably expected to last at least 12 months (unless death intervenes) and to involve either several different organ systems or 1 organ system severely enough to require specialty pediatric care and probably some period of hospitalization in a tertiary care center complex" (Feudtner et al, 2014).[20,21] The medical condition can vary from rare illnesses to premature birth, and organ transplants. The system used here identifies 9 categories (cardiovascular, respiratory, neuromuscular, renal, gastrointestinal, hematologic or immunologic, metabolic, other congenital or genetic, and malignancy).

© 2023 Koebnick C et al. JAMA Network Open.
