# Supplementary material for: Association of High Normal Body Weight in Youths With Risk of Hypertension
Source: JAMA Netw Open. 2023 Mar 14;6(3):e231987. doi: 10.1001/jamanetworkopen.2023.1987 (PMC10015306; doi:10.1001/jamanetworkopen.2023.1987)
Supplement: Supplement 2. — Data Sharing Statement [file jamanetwopen-e231987-s002.pdf]

Data Sharing Statement

Koebnick. Association of High Normal Body Weight in Youths With Risk of Hypertension. *JAMA Netw Open*. Published March 14, 2023. doi:10.1001/jamanetworkopen.2023.1987

Data
Data available: Yes
Data types: Deidentified participant data
How to access data: Anonymized data that support the findings of this study may be made available from the investigative team in the following conditions: (1) agreement to collaborate with the study team on all publications, (2) provision of external funding for administrative and investigator time necessary for this collaboration, (3) demonstration that the external investigative team is qualified and has documented evidence of training for human subjects protections, and (4) agreement to abide by the terms outlined in data use agreements between institutions.
When available: With publication

Supporting Documents
Document types: None

Additional Information
Who can access the data: Researchers who meet the following conditions: in the following conditions: (1) agreement to collaborate with the study team on all publications, (2) provision of external funding for administrative and investigator time necessary for this collaboration, (3) demonstration that the external investigative team is qualified and has documented evidence of training for human subjects protections, and (4) agreement to abide by the terms outlined in data use agreements between institutions.
Types of analyses: specified purpose
Mechanisms of data availability: Once conditions above are met and contract is in place.
